# Isolation and Characterization of Aquatic-Borne *Klebsiella pneumoniae* from Tropical Estuaries in Malaysia

**DOI:** 10.3390/ijerph13040426

**Published:** 2016-04-15

**Authors:** Anis Barati, Aziz Ghaderpour, Li Lee Chew, Chui Wei Bong, Kwai Lin Thong, Ving Ching Chong, Lay Ching Chai

**Affiliations:** 1 Faculty of Science, Institute of Biological Sciences, University of Malaya, Kuala Lumpur 50603, Malaysia; barati.anis@gmail.com (A.B.); ghaderpour_aziz@yahoo.com (A.G.); lileechew@um.edu.my (L.L.C.); cwbong@um.edu.my (C.W.B.); thongkl@um.edu.my (K.L.T.); chong@um.edu.my (V.C.C.); 2 Department of Biomedical Sciences, Wide River Institute of Immunology, Seoul National University College of Medicine, Seoul 03080, Korea; 3 Institute of Ocean & Earth Sciences, University of Malaya, Kuala Lumpur 50603, Malaysia; 4 Faculty of Applied Sciences, UCSI University, Kuala Lumpur 56000, Malaysia

**Keywords:** *Klebsiella pneumoniae*, aquatic, anthropogenic contamination, antimicrobial resistance, virulence factors

## Abstract

*Klebsiella pneumoniae* is an opportunistic pathogen that is responsible for causing nosocomial and community-acquired infections. Despite its common presence in soil and aquatic environments, the virulence potential of *K. pneumoniae* isolates of environmental origin is largely unknown. Hence, in this study, *K. pneumoniae* isolated from the estuarine waters and sediments of the Matang mangrove estuary were screened for potential virulence characteristics: antibiotic susceptibility, morphotype on Congo red agar, biofilm formation, presence of exopolysaccharide and capsule, possession of virulence genes (*fimH*, *magA*, *ugE*, *wabG* and *rmpA*) and their genomic fingerprints. A total of 55 strains of *K. pneumoniae* were isolated from both human-distributed sites (located along Sangga Besar River) and control sites (located along Selinsing River) where less human activity was observed, indicated that *K. pneumoniae* is ubiquitous in the environment. However, the detection of potentially virulent strains at the downstream of Kuala Sepetang village has suggested an anthropogenic contamination source. In conclusion, the findings from this study indicate that the Matang mangrove estuary could harbor potentially pathogenic *K. pneumoniae* with risk to public health. More studies are required to compare the environmental *K. pneumoniae* strains with the community-acquired *K. pneumoniae* strains.

## 1. Introduction

Bacteria of the *Klebsiella* genus are responsible for a variety of diseases in animals and humans [1]. Of the four disease-causing *Klebsiella* species, *K. pneumoniae* is the medically most important species as compared with *K. oxytoca*, *K. ozaenae* and *K. rhinoscleromatis*. *K. pneumoniae* has both clinical and non-clinical habitats [2]. Surface water, drinking water, soil, plants, sewage, and industrial effluent are the environmental reservoirs of *K. pneumoniae* [3,4]. In fact, due to its widespread nature, even in the environments apparently free from obvious fecal contamination, *K. pneumoniae* is usually considered as a member of total coliforms with insignificant public health risk [5]. However, Padschun and co-workers isolated potentially pathogenic *K. pneumoniae* from the aquatic environment in Germany that possess virulence factors such as pili, serum resistance and siderophore [6]. These virulence factors are commonly present in the clinical isolates which therefore suggest that the *K. pneumoniae* of environmental origin could have public health implications [7]. Moreover, *K*. *pneumoniae* is a common hospital-acquired pathogen that frequently causes nosocomial outbreaks. It can cause urinary tract, respiratory tract, soft tissue infections and liver abscess syndrome, which have been found to be a common source of infections particularly in Southeast Asia [8,9,10,11].

During the past two decades, highly virulent strains of *K. pneumoniae* have emerged as a predominant cause of pyogenic liver abscess (PLA). PLA is a potentially life-threatening disease due to complications of bacteremia, sepsis, and metastatic infection of brain, eyes, lungs and other organs, especially in patients with diabetes [12,13]. A number of virulence genes of *K. pneumoniae* were found to play major roles in the pathogenesis of PLA. Presence of *rmpA* gene (a regulator of the mucoid phenotype), *magA* gene (capsular polysaccharide synthesis) and K1 or K2 capsular serotype are the dominant characteristic of PLA strains [14]. Studies conducted across the world have revealed geographical differences and trends of the dominant *K. pneumoniae* strains causing the community-acquired infections [9,15]. The emergence of invasive PLA has been frequently reported in Southeast Asian countries, while in the United States, Argentina, Europe, or Australia PLA is less frequent [9]. The significant differences between Southeast Asia and the Western countries could be due to variation in the bacterial virulence and drug resistance pattern, as well as the host immunity and the antibiotic consumption [16].

The development of antibiotic resistance among hospital-acquired *K. pneumoniae* is a growing concern globally. The widespread use of broad-spectrum antibiotics in hospitals, agriculture, and aquaculture sectors could lead to antibiotic residues contamination of the aquatic environments, and eventually causes the emergence of community antibiotic-resistant *K. pneumoniae* [17]. Community-acquired *K. pneumoniae* frequently cause meningitis, pulmonary infections and liver abscesses in Southeast Asian countries [9], and thus, understanding the diversity of *K. pneumoniae* distributions in the estuarine environments in terms of their virulence characteristics, genetic background and antibiotic resistance will be of great interest.

In this study, the occurrence of *K. pneumoniae* in the Matang mangrove estuary was determined. The Matang mangrove estuary, which is located on the northwest coast of Peninsular Malaysia, is known as one of the best managed coastal forests in the world [18]. The Matang mangrove estuary is a habitat for various organisms such as fishes, shellfish, microbes and a vast variety of flora. Therefore, the water quality and particularly the microbial component of its estuarine system may affect the socio-economic activities in the estuary. Numerous fishing villages are located by the Matang mangrove estuary, with Kuala Sepetang, with a total population of 31,800 people, 79% of which are fishermen, as the biggest village [19]. Due to the heavy impact of human activities, including human settlements and aquaculture farming, anthropogenic pollution is significant in the Matang mangrove estuary, which is contaminated with various potentially pathogenic bacteria including *E. coli*, *K. pneumoniae*, *Serratia marcescens* and *Enterobacter cloacae* [19]. In view of the reported high incidence rate of diseases caused by *K. pneumoniae* in Southeast Asia [20], we investigated the diversity and virulence of *K. pneumoniae* isolated from the Matang mangrove estuary. In this study, the Matang mangrove estuary was studied for the occurrence of potentially pathogenic aquatic-borne *K. pneumoniae* by phenotype and genotype properties. Furthermore, the genetic diversity of isolated *K. pneumoniae* was determined by Repetitive Extragenic Palindromic PCR (REP-PCR).

## 2. Experimental Section

### 2.1. Sampling Location

**Figure 1:**
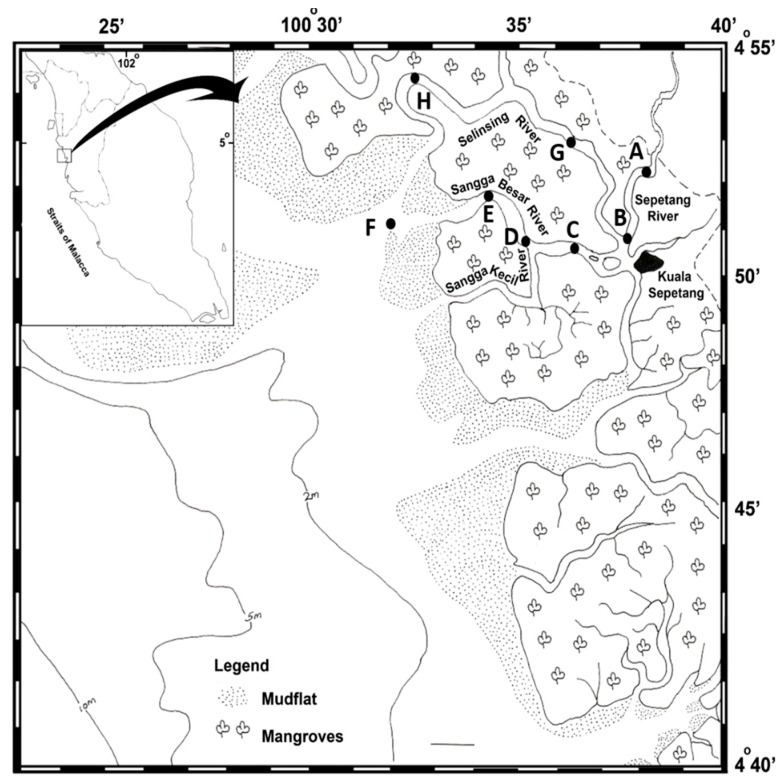
Locations of the eight sampling stations (**A**–**H**) along the Sepetang, Sangga Besar and Selinsing rivers in the Matang mangrove estuaries, Perak, Malaysia. **A**–**H**: Sampling stations along the Matang mangrove estuary, Perak, Malaysia.

Water and sediment samples were collected from eight stations located along the Sepetang, Sangga Besar and Selinsing rivers of the Matang mangrove estuary (Perak, Malaysia) in February 2012. The main waterway for fishing boats from Kuala Sepetang to the fishing grounds on the coast is the Sangga Besar River. The Sepetang River is located upstream and brings the majority of fluvial discharges downstream to the river mouth. Furthermore, a few fish cages are located at the river mouth. All the sampling stations were selected from upstream of the estuary to the coastal mudflat area (Figure 1). Station A was located upstream of the Sepetang River, and stations B and C were situated close to the Kuala Sepetang village. Stations D, E, and F were alongside the Sangga Besar river areas of cockle culture and floating cages respectively. Station G and H respectively from the upstream and downstream part of the Selinsing River were selected. Station H was located far from human activities and was included to contrast with station A–G.

### 2.2. Sample Collection

A total of thirty-two samples of both estuarine water (*n* = 16) and sediment (*n* = 16) were collected from eight sampling sites. The water samples were collected using a Van Dorn sampler (Wildco, Yulee, FL, USA) and were stored in acid-washed bottles. Surface sediment samples (0–5 cm) were collected by Ekman Grab (Wildco) and were placed into 50-mL sterile bottles. All samples were kept in an ice chest and were transferred immediately to the laboratory at 4 °C. Microbiological analyses were performed within 24 h. The *in-situ* parameters of water pH, salinity and temperature at each station were measured by a multi-parameter probe (565 MPS, YSI, Yellow Springs, OH, USA).

### 2.3. Detection and Isolation of K. pneumoniae

The presumptive *K. pneumoniae* were isolated from water and sediment samples using the spread plate method onto CHROMagar™ (DRG International, Inc., Springfield, NJ, USA). 5 mL and 10 mL of estuarine water samples from different sampling sites were filtered through 0.45 mm nitrocellulose filters (47 mm diameter). The filtrates were serially diluted with sterile saline (0.85% NaCl) before spread plating, whereas the concentrated filter membranes were transferred into 10 mL of sterile Buffer Peptone Water (BPW) and enriched at 37 °C for 24 h. The overnight enriched BPW were then sub-cultured onto CHROMagar™. For sediment samples, 1 gram of sediment was homogenized in 9 mL of sterile saline (0.85% NaCl). Five mL of the homogenate was then filtered through a 0.45 mm nitrocellulose filter (47 mm diameter) and serially diluted with sterile saline (0.85% NaCl) before spread plating. The presumptive bacterial colonies with metallic blue morphology on CHROMagar™ were picked and sub-cultured for further bacterial identification with standard biochemical assays (indole, Methyl Red, Voges-Proskauer, citrate utilization, sugar fermentation on triple sugar iron (TSI), gas production, motility, sulfur production, urea hydrolysis, oxidase and catalase) [21] or identification with the BIOLOG GenIII bacterial identification system (Biolog Inc., San Francisco, CA, USA) before subjected to PCR for confirmation. Randomly confirmed strains of *K. pneumoniae* were selected for 16S rDNA PCR-sequencing for double confirmation. All tests were performed in duplicate.

### 2.4. PCR Confirmation of K. pneumoniae

The confirmation of presumptive *K. pneumoniae* isolates was carried out to target the malate dehydrogenase (*mdh*) housekeeping gene [22]. The template DNA for PCR was prepared by boiled cell method [23] and then the PCR was carried out in a master mixture containing DNA Taq polymerase (0.5 U/mL), dNTPs (220 µM), MgCl_2_ (25 µM) and each primer (0.8 µM). The PCR condition was: 95 °C for 5 min, followed by 30 cycles of 95 °C for 1 min, 53 °C for 1 min, 72 °C for 1 min and 72 °C for 5 min (Table 1). Finally, the visualization of PCR amplicons was performed via horizontal agarose gel electrophoresis for 30 min at 100 V. Finally, three PCR products were randomly chosen for purification using HiYield Gel/PCR DNA fragments extraction kit (Yeastern Biotech Co., Ltd., New York, NY, USA) and then sequenced by a local service provider (First BASE Laboratories, Kuala Lumpur, Malaysia). The sequences of the amplified genes from the PCR reactions were compared with the GenBank databases of nr/nt using BLASTN [24] for validation purposes.

### 2.5. Detection of Virulence-Associated Genes

Four chromosome-mediated virulence genes, encoding for biosynthesis of outer core lipopolysaccharide (*wabG*), capsule and smooth lipopolysaccharide (*ugE*), type one fimbria adhesion (*fimH*), capsular polysaccharide synthesis (*magA*) and one plasmid-mediated gene, encoding for a regulator of the mucoid phenotype (*rmpA*) [25,26,27,28] were detected by PCR assays. Bacterial DNA was extracted via boiled cell method [23] and the primer pairs listed in Table 1 targeting specific virulence genes were used. The final PCR master mixture contained DNA *Taq* polymerase (0.5 U/mL), dNTPs (220 µM), MgCl_2_ (25 µM) and each primer (0.2 µM). The PCR conditions for *wabG*, *ugE* and *fimH* were: 95 °C for 5 min, followed by 30 cycles of 95 min for 1 min, appropriate annealing temperature for 45 seconds (Table 1), 72 °C for 1 min and 72 °C for 5 min. While the reaction mixture for *magA* and *rmpA* were kept at 95 °C for 5 min, followed by 40 cycles of 95 °C for 1 min, 50 °C for 1 min (Table 1), and 72 °C for 2 min, and 72 °C for 7 min. Finally, the PCR products from each group of genes were randomly chosen for purification using HiYield Gel/PCR DNA fragments extraction kit (Yeastern Biotech Co., Ltd., New York, NY, USA) and then sequenced by a local service provider (FirstBASE Laboratories, Kuala Lumpur, Malaysia). The sequences of the amplified genes from the PCR reactions were compared with the GenBank databases of nr/nt using BLASTN [24] for validation purposes.

### 2.6. Hemolytic Assay, Hyperviscosity Test and Capsule Detection

For the hemolytic activity assay, an overnight *K. pneumoniae* culture was performed on blood agar medium containing 5% (W/V) sheep blood as described by Gerhardt and co-workers. The inoculated plates were incubated for 24 h at 37 °C [31]. The hyperviscosity phenotype of the *K. pneumoniae* isolates was determined using a modified string test [32]. The formation of a viscous string of at least 1 cm was considered positive. The presence of a capsule in *K. pneumoniae* was determined by Maneval’s staining method [33]. Moreover, PCR was used for detection of K1 and K2 capsular serotype of *K. pneumoniae* [29]. The DNA template was extracted by boiling cell method [23] and the primer pairs listed in Table 1 were used to target the K1 and K2 capsular serotypes genes. PCRs were carried out in 25 μL volumes containing 5 μL extracted DNA, with 1.5 U of DNA *Taq* polymerase, 0.2 µM of dNTP, 3.0 µM of MgCl_2_, and 0.8 μM of each primer. Conditions were: 94 °C for 5 min, followed by 35 cycles of 94 °C for 45 s, 56 °C for 45 s, 72 °C for 45 s, and a final extension at 72 °C for 5 min. Finally, the PCR products from each group of genes were randomly chosen for purification using HiYield Gel/PCR DNA fragments extraction kit (Yeastern Biotech Co., Ltd., New York, NY, USA) and then sequenced by a local service provider (FirstBASE Laboratories, Kuala Lumpur, Malaysia). The sequences of the amplified genes from the PCR reactions were compared with the GenBank databases of nr/nt using BLASTN [24] for validation purposes.

### 2.7. Morphotypes on Congo Red Agar Plates

The morphotypes were checked by culturing on Luria broth agar without salt which was supplemented with 20 µg/mL of Comassie brilliant blue and 40 µg/mL of Congo red [34]. The *K. pneumoniae* isolates were subcultured and incubated at 28 °C [35] and 37 °C [36] for 96 h to determine bacterial colony colour and rugosity [37]. The categorization of morphotypes was based on Römling’s study [37]. The development of a red, dry and rough colony morphology (RDAR) on Congo red agar plates represented bacteria with cellulose production and the curli phenotype. Disruption of one or both of these incorporators created distinct colony morphology types. Deficiency in curli and cellulose synthesis causes a smooth and white (SAW) colony appearance, while defects in cellulose synthesis and curli expression leads to brown colonies (BDAR). A deficiency in curli expression results in pink colonies (PDAR) [37]. Bacterial expression of curli and cellulose has an important effect on biofilm production [38]. *Salmonella enterica* serovar typhimurium was used as a control for Congo Red Agar morphotype [39] and the test was performed in duplicate.

### 2.8. In Vitro Biofilm Formation Assay

*K. pneumoniae* isolates were sub-cultured three times in Luria broth (LB) for 18 h at 37 °C according to Kwasny and Opperman [40]. The optical density of the third bacterial suspension was adjusted to 0.56 to 0.64 (2.107 to 8.108 colony forming unit (CFU/mL)) at 540 nm. From the adjusted concentration of suspensions, 200 µL were transferred to 96 well polystyrene microtiter plates and incubated for 24 h at 37 °C. After that, the broth was removed and the wells were washed with PBS buffer and incubated for 1 h at 60 °C for biofilm fixation. Subsequently, the biofilms were stained with 0.06% crystal violet for 15 min, then the stains were discarded and the wells were washed with PBS buffer. Finally, the stained biofilms were dissolved in 95% ethanol and the optical density (OD) was read at 590 nm [41]. Different ranges of absorbance at 590 nm indicate different levels of biofilm formation. The strains were categorized as strong biofilm formers (OD: *x* > 1.51), weak biofilm formers (OD: 0.51 ≤ *x* ≤ 1.5) and non-biofilm formers (OD: *x* ≤ 0.5). The test was repeated five times.

### 2.9. Antibiotic Susceptibility Tests

Antibiotic susceptibility testing was performed using the disk diffusion method on Muller-Hinton agar according to the CLSI 2012 protocol [42]. The antibiotics tested were: amoxicillin/clavulanic acid (30 µg), ampicillin (10 µg), ceftiofur (30 µg), streptomycin (25 µg), kanamycin (30 µg), gentamicin (10 µg), trimethoprim-sulfamethoxazole (25 µg), chloramphenicol (30 µg), tetracycline (30 µg), oxolinic acid (2 µg), nalidixic acid (30 µg), ciprofloxacin (5 µg), enrofloxacin (5 µg), levofloxacin (5 µg), ceftazidime (30 µg), meropenem (10 µg), ertapenem (10 µg), cefepime (30 µg) and neomycin (30 µg) from Oxoid Ltd. (Basingtsoke, Hants, UK). *E coli* ATCC 25922 was used as a control. The multidrug resistance (MDR) status was noted for those isolates that were resistant to ≥3 antimicrobial categories. *K. pneumoniae* is intrinsically resistant to ampicillin, and, therefore, the result of this antibiotic was not included in the calculation of antibiotic resistance patterns. The test was performed in duplicate.

### 2.10. Repetitive Extragenic Palindromic PCR (REP-PCR)

REP-PCR was used on 55 aquatic-born *K. pneumoniae* isolates from the Matang mangrove estuary with the primer mentioned in Table 1 for the clustering of *K. pneumoniae*. The PCR conditions and gel electrophoresis were performed as described previously [43]. The REP-PCR products were analyzed on a 1.5% agarose gel. The gel electrophoresis was performed at 100 V for 6 h using 0.5% tris-borate EDTA, and then the gel was stained with GelRed™ (Biotium, Hayward, CA, USA) and visualized by Gel doc (Bio-Rad, Hercules, CA, USA).The dendrogram was constructed by Bionumerics version 6 (Applied Maths, Kortrijk, Belgium) based on the comparison of band patterns at 1.5% tolerance.

### 2.11. Statistical Analysis

Principle component analysis (PCA) was used to reveal the distribution of antibiotic resistant *K. pneumoniae* among the seven sampling stations in Matang mangrove estuary. Prevalence of the antibiotic susceptibility was determined using x/y × 100% equation, where x is defined as number of non-susceptible *K. pneumoniae* isolates to antibiotics, and y is total isolated number of *K. pneumoniae* from each station. To perform the PCA procedure, the abundance of antibiotics resistant *K. pneumoniae* was arcsine transformed to meet the parametric assumptions. PCA was performed using CANOCO 4.5 software [44].

## 3. Results and Discussion

### 3.1. Detection and Isolation of K. pneumoniae from Estuarine Water and Sediments

In this study, the total coliform count in estuarine water was ranged from 10 to 10^3^ CFU/100 mL. A total of fifteen samples (47%) of estuarine water and sediment were found to contain *K. pneumoniae*. Notably, *K. pneumoniae* was the only *Klebsiella* species detected and isolated from the Matang mangrove estuary. Out of 80 presumptive *Klebsiella* isolated from CHROMagar, 55 isolates (69%) were confirmed by biochemical assays and PCR targeting of the *mdh* gene [22] as *K. pneumoniae*. Using the Biolog GenIII bacterial identification system and 16-srDNA PCR-sequencing, those presumptive *Klebsiella* isolates that were confirmed as non-*K. pneumoniae* were later being identified as *Enterobacter* spp. and *Rauotella planticola*. Podschun and co-workers had reported the isolation of more than one species of *Klebsiella*, including *K. oxytoca* and *K. planticola* (recategorized as *Rauotella planticola*), with *K. pneumoniae* being the most predominant species in fresh water, brackish water, and salt water [6]. It is unclear if the negative detection of *Klebsiella* species other than *K. pneumoniae* in this work is attributed to a truly low density of the other *Klebsiella* species in the mangrove estuary or the inability of CHROMagar to isolate *Klebsiella* species other than *K. pneumoniae*. However, as demonstrated by Merlino and co-workers, *K. oxytoca* is capable of growing on CHROMagar, showing colony morphology of metallic blue without a pink halo, which is similar to *K. pneumoniae* [45].

A total of fifty-five isolates of *K. pneumoniae* were isolated from seven out of the eight stations along the Sepetang, Sangga Besar and Selinsing rivers of the Matang mangrove estuary. Isolation of *K. pneumoniae* from station A (located upstream of the Kuala Sepetang village) and station H (located approximately 15 km downstream of the village), which are far from human settlement, suggests that the estuarine environment could be serving as a natural reservoir for this opportunistic pathogen. This finding is in agreement with other studies, which found *Klebsiella* spp., including *K. pneumoniae*, to be ubiquitous in natural waters [3,4,46]. In this study, *K. pneumoniae* was largely isolated from the estuarine water (*n* = 48; 87%), while this opportunistic pathogen was only detected in the sediment samples at station B and D (*n* = 7; 13%). Station B was located immediately downstream of Kuala Sepetang village, thus receiving the heaviest pollution from the village. Station D was located within the cockle culture bed. The detection of *K. pneumoniae* from the sediment and water pose a concern of contamination of cockles with *K. pneumoniae*.

### 3.2. Virulence Factors and Phenotypic Characterization of Aquatic-Borne K. pneumoniae

Despite being known as a species naturally occurring in the environment, specifically in contaminated water, the virulence properties, genetic and phenotypic variations among the aquatic-borne *K. pneumoniae* remain largely unknown. Podschun and co-workers reported that the water-borne *K. pneumoniae* were similar to the clinical strains in term of their virulence properties [6]. In this study, all of the fifty-five isolates of aquatic-borne *K. pneumoniae* grew on blood agar as greyish colonies and displayed no hemolysis activity towards red blood cells, in agreement with the previous study by Szramka and co-workers [47]. Capsule is an important structure of *K. pneumoniae* that facilitates colonization and invasion of the human host. Struve and Krogfelt [48] reported that non-capsulated *K. pneumoniae* had reduced attachment to epithelial cells and also a reduction in pathogenicity. Almost all clinical strains of *K. pneumoniae* possess capsule and so does the environmental isolates, as reported by Podschun and co-workers [6]. We detected capsule in all (*n* = 53; 96%) except two isolates from station B (sediment sample) and G, located upstream of the estuary. These two non-encapsulated isolates were also negative for the five virulence genes (*fimH*, *wabG*, *ugE*, *magA* and *rmpA*) and were weak biofilm-formers. The results suggest that both of the non-encapsulated *K. pneumoniae* isolates are not likely to be pathogenic. Furthermore, PCR assay for the K1 and K2 serotypes was detected just the K2 serotype for six isolates that originated from station B, which was located immediately downstream of the Kuala Sepetang village. Hyperviscosity is another important surface-related virulence factor of *K. pneumoniae* and is specifically essential for invasive PLA [14]. Out of the fifty-five isolates examined, five (9%) isolates demonstrated hyperviscosity (Table 2). Interestingly, out of these five hyperviscous *K. pneumoniae* isolates, three of these isolates were located at station B which were K2 serotypes.

In this study, a number of virulence genes related to bacterial surface properties of *K. pneumoniae* were screened: *wabG* (involved in biosynthesis of the outer core lipopolysaccharide), *ugE* (synthesis of UDP galacturonate 4-epimerase, which is responsible for biosynthesis of the capsule and smooth lipopolysaccharide), *fimH* (fimbrial gene encoding the adhesive subunit of type 1 fimbriae), *magA* (capsular polysaccharide synthesis) and *rmpA* (regulator of the mucoid phenotype) [25,26,27,28]. *magA* and *rmpA* virulence genes were not detected in all of the aquatic-borne *K. pneumoniae* isolates; while, *wabG*, *ugE* and *fimH* genes were detected in some of the isolates. The results indicated that half of the tested isolates (28/55; 51%) were positive for all *wabG*, *ugE* and *fimH* genes. Although *magA* and *rmpA* genes have high prevalence in the *K. pneumoniae* isolates from PLA, serotype K1 or K2 is the major virulence determinant for liver abscess caused by *K. pneumoniae*. It was previously reported that the higher virulence and higher resistance to phagocytosis render serotype K1 or K2 strains more likely to cause metastatic septic complications [28]. Hence, the detection of K2 serotype at the downstream of Kuala Sepetang village highlights the need to further study the potential public health risks attributed to the community-acquired infection. Additionally, 39 (70.9%) were positive for *fimH*, 47 (85%) for *wabG* and 39 (71%) for *ugE* (Table 2). Although very few *K. pneumoniae* were isolated from station E and F, all of the isolates from these two stations possessed all of the virulence genes (Table 2). Because *wabG*, *ugE* and *fimH* genes are related to the bacterial surface structure, it was speculated that the capsule, LPS and fimbriae play an important putative role of survival in water with high salinity, such as at stations E and F.

**Figure 2:**
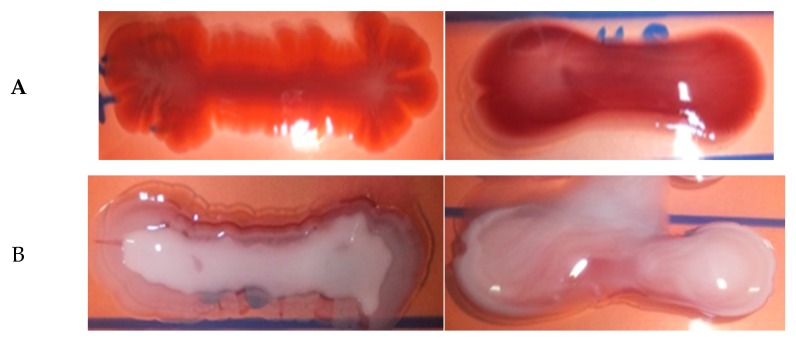
(**A**) From left to right: RDAR and PDAR phenotype at 37 °C after 72 h incubation; (**B**) From left to right: rough edge of RDAR phenotype and smooth shape of PDAR phenotype at 28 °C after 72 h incubation.

To further study the role of bacterial surface properties on survival in the estuarine environment, Congo red binding assay was conducted. Congo red binding is commonly used to study the surface properties of environmental bacteria. Several studies have reported that Congo red binding can be used to indicate the presence of bacterial extracellular matrix that contributes to pathogenicity [49,50]. In this study, it was found that all of the isolates were able to bind to the Congo red, demonstrating 71% red and rough (RDAR) and 29% pink and smooth (PDAR) morphotypes (Table 2; Figure 2). Sonbol and co-workers [51] also observed a very high prevalence of Congo red positive morphotypes in clinical strains. On the other hand, Zogaj *et al.* [38], reported negative result for Congo red binding in *Klebsiella* spp. isolated from healthy human gastrointestinal tracts. It should be noted that this method is not always accurate, as it sometimes gives false negative results, and therefore, it might be useful just for a preliminary pathogenicity screening [52]. Some studies had demonstrated that the biofilm formation ability was related to Congo red morphotypes [53]. Both RDAR and PDAR phenotypes were positive for extracellular matrix and cellulose production, but the PDAR phenotype showed lower levels of biofilm formation than RDAR [53]. Similarly, we observed a lower level of biofilm formation in the PDAR morphotype compared to the RDAR morphotype, although the difference was not statistically significant (Z = −0.834; *p* > 0.05). This could be due to the small sample size of PDAR morphotypes. However, it is noteworthy to point out that all of the strong biofilm former, hyperoviscous strains and K2 serotypes showed RDAR morphology.

### 3.3. Biofilm Production

Another important virulence factor of *K. pneumoniae* is its ability to form biofilms. Biofilm-forming *K. pneumoniae* have been associated with urinary tract infections and hospital-acquired pneumonia [54,55]. Yang and Zhang [56] demonstrated that approximately 40% of *K. pneumoniae* isolated from urine, sputum, blood and wound swabs were able to produce biofilm. Biofilm formation does not only confer survivability to the pathogen in the human hosts, but also persistence in the fluctuating environment such as variable pH, temperature, carbon sources, and fluid flow [57]. In this study, as many as 76.4% (*n* = 42) of the aquatic-borne *K. pneumoniae* isolates demonstrated biofilm-forming ability in the *in vitro* assay (Table 3). Isolates from station E and F that were characterized by higher water salinity (~23 ppt) demonstrated moderate to strong biofilm-forming ability while isolates from sediment sample showed weak biofilm forming ability. It seems that biofilm-forming ability is an important survival characteristic under high salinity. Others have reported that hyperviscous *K. pneumoniae* frequently produces biofilm [58,59]. Our study suggested that the biofilm-forming ability seems to be associated to the virulence as the non-biofilm forming isolates were often negative for the tested virulence genes, hyperviscosity and capsule production; while the strong biofilm forming isolates demonstrated the RDAR and hyperviscose morphology and K1 serotype.

### 3.4. Antibiotic Susceptibility Test

**Figure 3:**
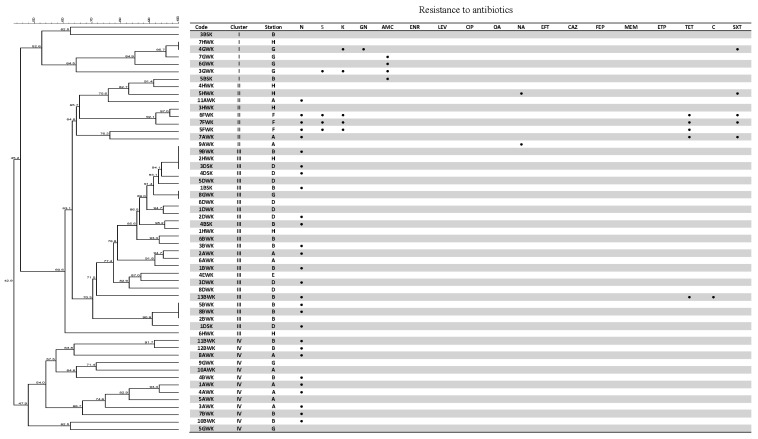
Dendrogram and table representing the resistance of *K. pneumoniae* isolates to the 19 types of antibiotics (N, neomycin; S, streptomycin; K, kanamycin; GN, gentamicin; AMC, amoxicillin/clavulanic acid; ENR, enrofloxacin; LEV, levofloxacin; CIP, ciprofloxacin; OA, oxolinic acid; NA, nalidixic acid; EFT, ceftiofur; CAZ, ceftazidime; FEP, cefepime; MEM, meropenem; ETP, ertapenem; TET, tetracycline; C, chloramphenicol; SXT, trimethoprim-sulfametoxazol). The dendrogram was constructed using the Bionumerics version 6 software.

Carbapenem-resistant *K. pneumoniae* has caused high morbidity and mortality in the long-term care-associated infections in hospitals [60]. Although carbapenemase-producing *K. pneumoniae* are spreading globally, in Malaysia there are no reports of these bacteria in hospitals or communities [61]. Several studies have reported the presence of multidrug-resistance (MDR), fluoroquinolone-resistance and extended-spectrum *β*-lactamase (ESBL)-producing *K. pneumoniae* in both clinical [43,62,63] and non-clinical isolates [64] in Malaysia. The aquatic-borne *K. pneumoniae* isolates from the Matang mangrove estuary were generally found to be susceptible to the antibiotics tested (Figure 3). However, a certain number of isolates demonstrating resistance towards multiple antibiotics were also detected, mainly from stations located immediately downstream of the villages (stations B, F, and G), indicating anthropogenic contamination as a potential source for introducing antibiotic resistance into the estuary.

**Figure 4:**
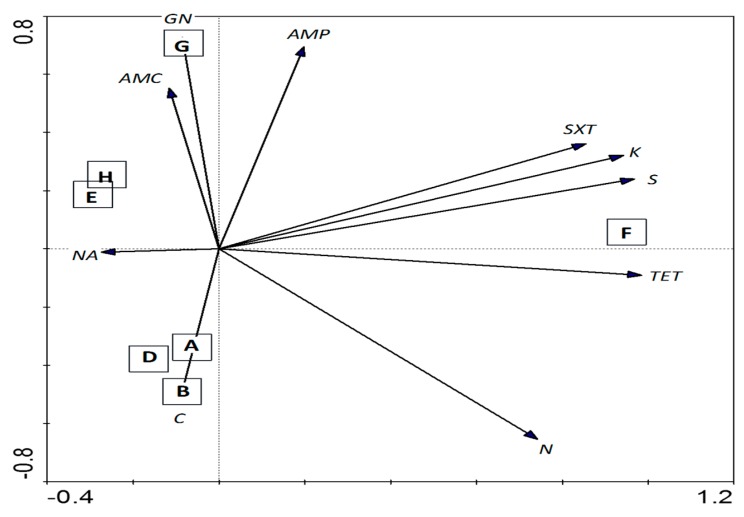
PCA ordination biplot showing the distribution of waterborne *K. pneumoniae* by sampling stations with respect to their susceptibility to various antibiotics. The first (PCA1) and second (PCA2) principal components respectively explained 74.2% and 17.3% of the total variance of the explanatory variables. The Capital letter in the square box denotes sampling stations located in Sepetang River (**A**), Sangga Besar River (**B**–**E**), Selinsing River (**G** and **H**) and adjacent coastal water (**F**). Black solid arrows indicate isolates demonstrating non-susceptibility towards 10 antibiotics: GN-NS, gentamicin; K-NS, kanamycin; S-NS, streptomycin; N-NS, neomycin; AMC-NS, amoxicillin/clavulanic acid; AMP-NS, ampicillin; NA-NS, nalidixic acid; TE-NS, tetracycline; SXT, sulfamethoxazole-trimethoprim; C, chloramphenicol.

Principle Component Analysis (PCA) showed that chloramphenicol resistance among the *K. pneumoniae* isolates was detected at the stations (A, B and D) near to the fishing village (Figure 4). Most *K. pneumoniae* isolated from these stations were of RDAR morphotypes and clustered together (cluster III) based on REP-PCR. Amoxicillin/clavulanic acid and gentamicin resistance were also detected in *K. pneumoniae* isolates from station G located along the Selinsing River (Figure 4). Station F which was located at the river mouth showed interesting particularities; associated to kanamycin, streptomycin, tetracycline and sulfamethoxazole-trimethoprim resistance (Figure 4); were resistant to more than five antibiotics (Figure 3) and were also moderate to strong biofilm-formers (Table 3). It is suggested that antibiotic resistance and the biofilm-forming ability of *K. pneumoniae* are somehow related. Several studies have reported higher antibiotic resistance among the biofilm-producing *K. pneumoniae* strains compared to the non-biofilm-producing strains [56,65,66]. The other four MDR isolates originated from stations B and G, located immediately downstream of Kuala Sepetang village. More surveillance is needed to assess the potential impact of anthropogenic contamination on the emergence of MDR pathogens from the aquatic environment.

### 3.5. Genetic Fingerprinting

REP-PCR fingerprinting demonstrated a high genetic diversity among the aquatic-borne *K*. *pneumoniae* isolates from the Matang mangrove estuary, with 48 REP-PCR genotypes detected (Figure 3). The clustering analysis grouped the 55 *K. pneumoniae* isolates into four clusters (I to IV). It was found that the antibiotic-sensitive isolates from stations A, B, and G, which were located close to each other and upstream of the estuary, are grouped together in cluster IV at a similarity level of 47.3%. Antibiotic-resistant isolates, with resistance specifically to nalidixic acid, trimethoprim, streptomycin and kanamycin, are grouped together in cluster II, at a similarity level of 64.6% (Figure 3). This study demonstrated that REP-PCR could be a good rapid fingerprinting method to type *K. pneumoniae* isolates from the aquatic environment.

## 4. Conclusions

The results suggested that *K. pneumoniae* strains isolated from the Matang mangrove estuary could be potentially virulent to humans based on the phenotypic and genotypic characterization. A number of virulence genes related to surface antigens which are responsible for pathogenicity were detected among isolates. The majority of the *K. pneumoniae* isolates (96.4%) were able to produce polysaccharide capsule, which is an important bacterial virulence factor. Moreover, a number of isolates were identified to be K2 capsular serotype, which is one of the most significant virulence factors. Additionally, biofilm formation was observed in about three quarters (76%) of the isolates. The isolates produced only two out of the four Congo red morphotypes (RDAR and PDAR), indicating variation in cellulose production and the curli phenotype. This study indicates that the Matang mangrove estuary harbors potentially virulent *K. pneumoniae* that poses a potential risk to public health. The potentially pathogenic *K. pneumoniae* in the estuary are likely linked to anthropogenic contamination. More studies and monitoring are needed to verify pathogenicity, public health risk and its occurrence or transmission to humans via seafood.

## Figures and Tables

**Table 1:** List of primers utilized in this study.

Gene	Primer	Sequence	Bp	Annealing Temp	MgC2	Ref
*Mdh*	*Mdh-f*	5′-GCGTGGCGGTAGATCTAAGTCATA-3′	364	53	1 μL	[22]
	*Mdh-r*	5′-TTCAGCTCCGCCACAAAGGTA-3′				
*wabG*	*wabG-f*	5′-ACCATCGGCCATTTGATAGA-3′	683	55	0.5 μL	[25]
	*wabG-r*	5′-CGGACTGGCAGATCCATATC-3′				
*ugE*	*ugE-f*	5′-TCTTCACGCCTTCCTTCACT-3′	535	56	1.5 μL	[26]
	*ugE-r*	5′-GATCATCCGGTCTCCCTGTA-3′				
*FimH*	*fimH-f*	5′-TGCTGCTGGGCTGGTCGATG-3′	550	58	1.5 μL	[27]
	*fimH-r*	5′-GGGAGGGTGACGGTGACATC-3′				
*magA*	*magA-f*	5′-GGTGCTCTTTACATCATTGC-3′	1282	50	1 μL	[28]
	*magA-r*	5′-GCAATGGCCATTTGCGTTAG-3′				
*rmpA*	*rmpA-f*	5′-ACTGGGCTACCTCTGCTTCA-3′	535	50	1 μL	[28]
	*rmpA-r*	5′-CTTGCATGAGCCATCTTTCA-3′				
K1	K1-f	5′-GTAGGTATTGCAAGCCATGC-3′	1046	56	3.0 μL	[29]
	K2-r	5′-GCCCAGGTTAATGAATCCGT-3′				
K2	K2-f	5′-GGAGCCATTTGAATTCGGTG-3′	1121	56	3.0 μL	[29]
	K2-r	5′-TCCCTAGCACTGGCTTAAGT-3′				
*Rep*	*Rep*	5′-GCGCCGICATGCGGCATT-3′	variable	44	2.5 μL	[30]

**Table 2:** Virulence properties of 55 isolated *K. pneumoniae* from the Matang mangrove estuary, Perak, Malaysia.

Station	No. of Isolates	Capsule	K2	Hyperviscosity	PDAR Morphotype	RDAR Morphotype	*fimH*	*ugE*	*wabG*
		No. (%)	Serotype	No. (%)	No. (%)	No. (%)	No. (%)	No. (%)	No. (%)
**A**	11	11 (100.0)	0	0	2 (18.18)	9 (81.82)	7 (63.6)	11 (100.0)	11 (100.0)
**B**	17	16 (94.1)	6 (35.29)	4 (23.5)	5 (29.41)	12 (70.59)	12 (70.6)	14 (82.3)	15 (88.2)
C *	0	0	0	0	0	0	0	0	0
**D**	9	9 (100.0)	0	0	2 (22.22)	7 (77.78)	5 (55.5)	4 (44.4)	8 (88.8)
**E**	1	1 (100.0)	0	0	0	1 (100)	1 (100.0)	1 (100.0)	1 (100.0)
**F**	3	3 (100.0)	0	0	2 (66.66)	1 (33.34)	3 (100.0)	3 (100.0)	3 (100.0)
**G**	7	6 (85.7)	0	0	2 (28.57)	5 (71.43)	5 (71.4)	2 (28.6)	4 (57.1)
**H**	7	7 (100.0)	0	1 (14.3)	3 (42.85)	4 (57.15)	6 (85.7)	4 (57.1)	5 (71.4)
**Total**	**55**	**53 (96.36)**	**6 (10.90)**	**5 (9.1)**	**16 (29.1)**	**39 (70.9)**	**39 (70.9)**	**39 (70.9)**	**47 (85.4)**

* *K. pneumoiniae* was not detected and isolated from station C.

**Table 3:** Biofilm formation capabilities among 55 isolated *K. pneumoniae* from seven sampling sites with different salinity from the Matang mangrove estuary (Perak, Malaysia).

Sampling Sites	Salinity	No. of Isolates (%)	No. of Non-Biofilm Formers (%)	No. of Weak Biofilm Formers (%)	No. of Moderate Biofilm Formers (%)	No. of Strong Biofilm Formers (%)
A	18.5	11	1 (9.1)	4 (36.4)	1 (9.1)	5 (45.4)
B	19.2	17	7 (41.2)	4 (23.5)	3 (17.6)	3 (17.6)
D	20.1	9	1 (11.1)	4 (44.4)	4 (44.4)	0
E	23.9	1	0	0	0	1 (100.0)
F	23.5	3	0	0	3 (100.0)	0
G	16.8	7	4 (57.1)	3 (42.9)	0	0
H	18.6	7	0	4 (57.1)	0	3 (42.8)
TOTAL		55	13 (23.6)	19 (34.5)	11 (20.0)	12 (21.8)
